# Illness perception in tuberculosis by implementation of the Brief Illness Perception Questionnaire – a TBNET study

**DOI:** 10.1186/2193-1801-3-664

**Published:** 2014-11-08

**Authors:** Dragica P Pesut, Bogdana N Bursuc, Milica V Bulajic, Ivan Solovic, Katarzyna Kruczak, Raquel Duarte, Adriana Sorete-Arbore, Marinela Raileanu, Irina Strambu, Ljudmila Nagorni-Obradovic, Tatjana Adzic, Zorica Lazic, Maria Zlatev-Ionescu, Sorokhaibam Bhagyabati, Irom Ibungo Singh, Govind Narayan Srivastava

**Affiliations:** Internal Medicine Department, University of Belgrade School of Medicine, Dr Subotica 8, 11000 Belgrade, Serbia; Clinical Centre of Serbia, Teaching Hospital of Lung Diseases, Koste Todorovica 26, Belgrade, Serbia; Department of Psychotherapy, Mind Institute, Bucharest, Romania; Faculty of Organizational Sciences Lab of Statistics, University of Belgrade, Jove Ilica 153, 11000 Belgrade, Serbia; Catholic University, Ruzomberk, Slovakia; National Institute for TB, Lung Diseases and Thoracic Surgery, Vysne Hagy, Kragujevac, Slovakia; Jagiellonian University School of Medicine, Kracow, Poland; University of Porto School of Medicine; Chest Disease Centre, Vila Nova de Gaia, Portugal; Hospital of Lung Diseases and TB, Iasi, Romania; Institute of Pneumology “Marius Nasta”, Bucharest, Romania; Department of Lung Diseases, University Centre Kragujevac, Kragujevac, Serbia; Clinical Hospital of Infectious Diseases “Dr. V. Babes”, Pulmonary Diseases, Bucharest, Romania; Regional Research Medical Centre, Manipur, India; Baranas Hindu University, Varanasi, India

**Keywords:** Tuberculosis, Illness perception, Questionnaire, Brief Illness Perception Questionnaire (BIPQ), Tobacco smoking

## Abstract

How patients relate to the experience of their illness has a direct impact over their behavior**.** We aimed to assess illness perception in patients with pulmonary tuberculosis (TB) by means of the Brief Illness Perception Questionnaire (BIPQ) in correlation with patients’ demographic features and clinical TB score.

Our observational questionnaire based study included series of consecutive TB patients enrolled in several countries from October 2008 to January 2011 with 167 valid questionnaires analyzed. Each BIPQ item assessed one dimension of illness perceptions like the consequences, timeline, personal control, treatment control, identity, coherence, emotional representation and concern. An open question referred to the main causes of TB in each patient’s opinion.

The over-all BIPQ score (36.25 ± 11.054) was in concordance with the clinical TB score (p ≤ 0.001). TB patients believed in the treatment (the highest item-related score for treatment control) but were unsure about the illness identity. Illness understanding and the clinical TB score were negatively correlated (p < 0.01). Only 25% of the participants stated bacteria or TB contact as the first ranked cause of the illness.

For routine clinical practice implementation of the BIPQ is convenient for obtaining fast and easy assessment of illness perception with potential utility in intervention design. This time saving effective personalized approach may improve communication with TB patients and contribute to better behavioral strategies in disease control.

**Electronic supplementary material:**

The online version of this article (doi:10.1186/2193-1801-3-664) contains supplementary material, which is available to authorized users.

## Background

Individuals diagnosed with an illness develop cognitive models to make sense of their ailment. These perceptions are important in guiding coping strategies and illness-specific behaviors (Broadbent et al. 2006; Petrie et al. 2002; Petrie et al. 2003). Patients may develop specific ideas about their disease. Research on illness perceptions may reveal differences between the physician’s view and the patient’s view, understanding and reaction (Bean et al. 2007). Changing patients’ illness perceptions is possible and it has been shown to improve recovery following myocardial infarction, and other self regulatory interventions in conditions such as diabetes mellitus (Petrie et al. 2002). In AIDS, the changes have improved patient outcome (Petrie et al. 2003).

Tuberculosis (TB) is a major cause of mortality and morbidity worldwide, affecting different countries disproportionately (World Health Organization 2011, 2014). Increasing number of HIV-infected people and the emergence of drug-resistant strains of *M. tuberculosis*, especially in Eastern Europe, make TB control more complicated (Migliori et al. 2008; Raviglione and Smith 2007; Jordan and Davies 2010; Stop TB Partnership and World Health Organization 2014). Proper information for patients and adherence to treatment are especially important in preventing drug resistance (Stop TB Partnership and World Health Organization 2014; WHO 2010; World Health Organization 2003). Recently, attempts have been made to identify the TB cases needing close follow-up to predict unsuccessful treatment outcome (Hasker et al. 2008; Baussano et al. 2008). Other studies have revealed that poor communication between health care staff and TB patients was a key issue underlying several causes of default and that TB patients lack proper information about this disease and its treatment (Hasker et al. 2010). Since some patients’ reactions are not fully explained by lack of information, they could be explained by illness perception, so assessment of this is warranted in routine clinical practice. Behavioral strategies are required for successful TB control. Ongoing research over the past 30 years has demonstrated the importance of illness representations to patients’ behavior (Broadbent et al. 2006; Petrie et al. 2002; Petrie et al. 2003). Perceptions can now be assessed by a number of psychometric instruments like the Brief Illness Perception Questionnaire (BIPQ), which has not been used for TB yet.

## Methods

### Study design and subjects

We aimed to assess illness perception in patients with pulmonary TB by implementation of the Brief Illness Perception Questionnaire (BIPQ) in correlation with the patients’ demographic data, social factors and clinical score at two time points: i) at the start of treatment, ii) at the end of the initial phase of treatment (after 2 months).

This observational study included a series of 178 consecutive newly diagnosed pulmonary TB patients (World Health Organization 2003) aged 18 years or older enrolled at TB hospitals in Europe and Asia between 1 October 2008 and 31 January 2011.

Data were collected by members of the Tuberculosis Network European Trials Group (TBNET) and allied researchers from Poland, Portugal, Romania, Serbia, Slovakia and India, who entered them in Microsoft Excel worksheets. The ethical board of the coordinating centre at the University Clinical Centre of Serbia, Belgrade, approved the study (12/4 B).

### Questionnaires

The patients’ clinical questionnaire was created to obtain demographic (sex, age) and social (marital status, profession, education) factors, tobacco smoking status and TB score (the components of the latter are listed in Table 1). The other one was the original Brief Illness Perception Questionnaire (BIPQ). After giving informed consent, all the 178 patients voluntarily completed the questionnaires at the start of treatment (0-month) for demographic and social data, symptoms as part of a TB score and the BIPQ. Medical staff were involved in providing relevant clinical data related to the TB score. The 167/178 questionnaires were valid, and 93/167 patients were retested after 2-months.

**Table 1:** **TB score components at the beginning of treatment (0) and after two months at the end of the continual phase of therapy (2)**

	TB score item	0 (N _0_ = 167)		0 (N _0_ = 93)		2 (N _2_ = 93)	
		N°	%	N°	%	N°	%
1	Cough	133	79.64	73	78.49	55	59.14
2	Hemoptysis	35	20.96	17	18.28	7	7.53
3	Dyspnea	67	40.12	35	37.63	26	27.96
4	Chest pain	61	36.53	31	33.33	15	16.13
5	Night sweats	93	55.69	45	48.39	23	24.73
6	Anemic	56	33.53	27	29.03	13	13.98
7	Tachycardia	62	37.13	31	33.33	15	16.13
8	Auscultation	99	59.28	34	36.56	71	76.34
9	Temperature > 37°C	82	49.10	44	47.31	9	9.68
10	BMI < 18	44	26.35	20	21.51	15	16.13
11	BMI < 16	22	13.17	5	5.37	2	2.15
12	MUAC < 220	55	32.93	19	20.43	20	21.51
13	MUAC < 200	22	13.17	6	6.45	2	2.15

BMI = Body Mass Index; MUAC = Mid Upper Arm Circumference.

### TB score

A simple clinical score was developed by Wejse et al. ( 2008) for repeated clinical status evaluation of TB patients during treatment without using advanced technical equipment. It is a useful clinical index, which is sensitive to changes during treatment. TB score components included self reported symptoms (cough, dyspnea, night sweats, hemoptysis and chest pain) and signs (anemia: paleness of conjunctivae at eye-examination; tachycardia: pulse rate ≥90/min; positive finding at lung auscultation: any one of the following findings present: crepitation, rhonci, subdued or complete absence of respiratory sounds; axillary temperature: temperature ≥37.08°C measured by an electronic thermometer in a closed axillary fold; body mass index (BMI): height measured using a meter scale and weight determined at each visit using the same balance. BMI = weight/(height)^2^; mid upper arm circumference (MUAC): measured over biceps of the non-dominant arm with a non-stretchable measuring tape. We used original instructions to score (the higher the score, the more severe clinical form of TB) (Wejse et al. 2008) and perform statistical analysis.

### BIPQ

The BIPQ is a 9-item questionnaire, used to measure illness perceptions along the following dimensions: identity, consequences, timeline, personal control, treatment control, concern, understanding and emotional representations (Broadbent et al. 2006). Each dimension is measured as a single item scored on an 11-point Likert scale, with higher scores indicating stronger endorsement of that item. According to the original instructions, summary score was also calculated by adding all of the BIPQ individual items to reflect the overall positivity or negativity of an individual’s illness perceptions. Finally, the BIPQ included an open question aimed to assess patients’ opinion about the three main causes of their disease in a rank order (Broadbent et al. 2006). The BIPQ is presented in Additional file 1.

The BIPQ forward and back-translation process caused no difficulties. After linguistic validation, BIPQ versions conceptually and linguistically equivalent to the original instrument were offered to the patients in their native languages. Implementation of the BIPQ in research on renal disease, type 2 diabetes mellitus, myocardial infarction, asthma, and minor disturbances showed good test retest validity (Broadbent et al. 2006) and another study led to its intercultural validation (Bean et al. 2007). The calculated minimum number of study group participants is 85 (Broadbent et al. 2006).

### Data analysis

Data were entered in Microsoft Excel worksheets and IBM SPSS Statistics 19 was employed for the analysis. We used original instructions to score the BIPQ (Broadbent et al. 2006) and TB score (Wejse et al. 2008). Bivariate correlations among the clinical and BIPQ scores at different time points were examined using Pearson’s Correlation Coefficient (r). The existence of significant differences between clinical and BIPQ scores at different time points was tested by corresponding paired t-tests. The significance levels were set at 0.01 < p ≤ 0.05 (statistically significant) and p ≤ 0.01 (highly statistically significant).

## Results

**Figure 1:**
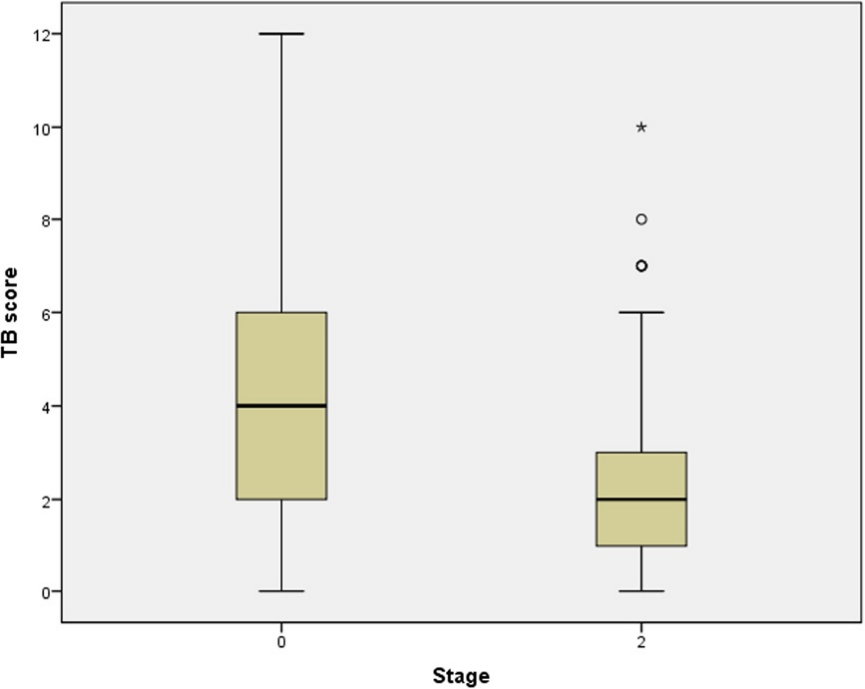
**The mean clinical scores of TB patients (TBscore) at two time points: 0 - at the start of treatment and 2 - at the end of the initial phase show significant difference; paired t-test (p < 0.001) N = 93.**

The total of 167/178 patients with pulmonary TB and valid questionnaires consisted of 104 (62.3%) men and 63 (37.7%) women of mean age 43.57 ± 14.462 years (18–83, range). The mean clinical score (TBscore) was 4.79 ± 2.918 in 167 patients’ sample at the beginning of treatment. In the sample of 93/167 patients retested at 2-month point the scores were 4.16 ± 2.871 and 2.41 ± 2.285 at time points 0 and 2, respectively (Figure 1), and the findings were highly significantly different (paired t-test, p < 0.001). The components of TB score results are shown in Table 1.

**Figure 2:**
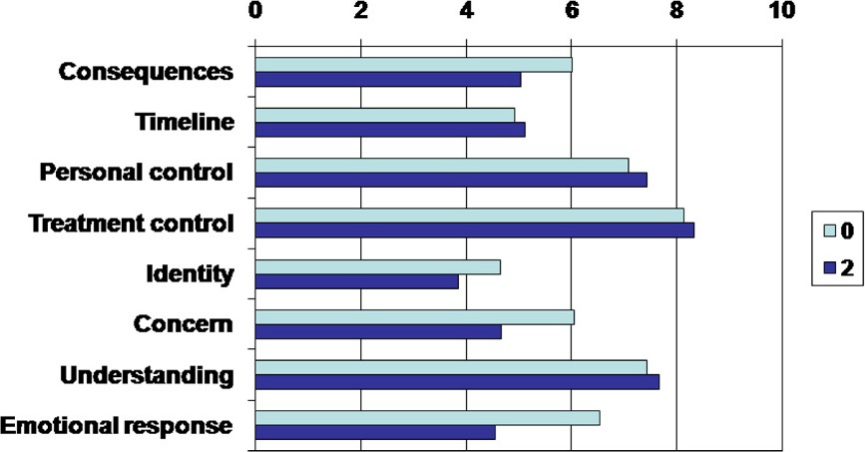
**The mean values of BIPQ item scores at the start of anti-tuberculosis therapy (0) and at the end of the initial phase of treatment (2) N = 93.**

Total mean BIPQ score was 36.25 ± 11.053 at the start of treatment. We found close positive correlations between total mean BIPQ and TB scores at both time points (p < 0.001, 2-tailed). Mean values for the BIPQ items and their correlation with clinical score are presented in Table 2. The highest BIPQ item-related score was found for treatment control and the lowest for timeline (illness duration) and identity (Tables 2 and 3, and Figure 2).

**Table 2:** **Mean values of BIPQ items and correlation with clinical TB score at the start of treatment**

BIPQ items	Mean value ± SD	Correlation	Significance (p)
1. Consequences	5.96 ± 3.144	0.072	
2. Timeline	4.82 ± 2.775	0.103	
3. Personal control	6.95 ± 2.780	-0.054	
4. Treatment control	8.26 ± 2.464	-0.108	
5. Identity	4.86 ± 2.877	0.268	<0.001
6. Concern	6.44 ± 2.949	0.084	
7. Understanding	7.38 ± 2.751	-0.215	<0.001
8. Emotional response	6.62 ± 2.932	0.100	
Total BIPQ score	36.25 ± 11.054	0.271	<0.001

BIPQ = Brief Illness Perception Questionnaire (Broadbent et al. 2006); TBscore (Wejse et al. 2008).

The p-values are entered only where the correlation is significant.

**Table 3:** **The mean values of BIPQ item scores at the start of anti-tuberculosis therapy (0) and at the end of the initial phase of treatment (2)**

BIPQ item	Mean value ± SD	Mean value ± SD	Mean value ± SD
	(0)	(0)	(2)
	N = 167	N = 93	N = 93
1. Consequences^#^	5.96 ± 3.144	6.01 ± 2.958	5.03 ± 3.171
2. Timeline	4.82 ± 2.775	4.92 ± 2.700	5.12 ± 2.532
3. Personal control	6.95 ± 2.780	7.09 ± 2.831	7.43 ± 2.939
4. Treatment control	8.26 ± 2.464	8.13 ± 2.950	8.32 ± 2.675 max
5. Identity^#^	4.86 ± 2.877	4.65 ± 2.842	3.85 ± 2.596
6. Concern^#^	6.44 ± 2.949	6.05 ± 2.983	4.66 ± 2.865
7. Understanding	7.38 ± 2.751	7.44 ± 2.984	7.66 ± 2.947
8. Emotional response^#^	6.62 ± 2.932	6.54 ± 2.865	4.55 ± 3.070 min
**Total BIPQ score** ^**#**^	**36.25 ± 11.054**	**35.6237 ± 11.15203**	**29.795 ± 13.277**

^#^Significant difference between 0- and 2-month point (p < 0.01).

**Figure 3:**
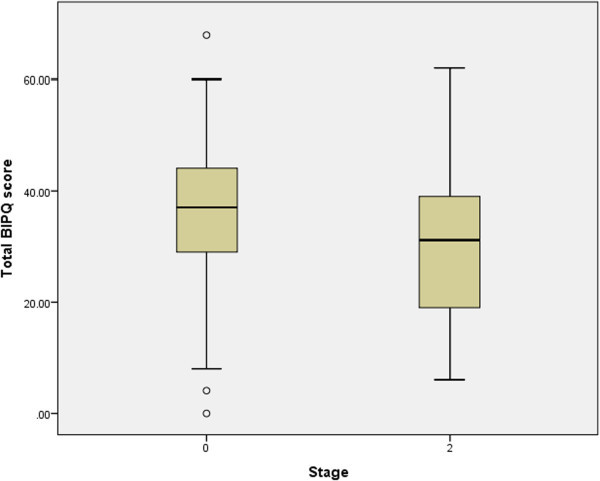
**Total BIPQ scores of TB patients at two time points: 0 - at the start of treatment and 2- at the end of the initial phase N = 93.**

We found a significant difference between mean values for total BIPQ score at the beginning of treatment (0-month point) and the total score at the end of the initial phase of therapy (2-month point); paired samples test, 2-tailed, p < 0.001 (Table 3). Total BIPQ scores at both time points are shown in Figure 3 for 93 patients.

The answers to the BIPQ open question on the main causes of the illness by patients’ own opinion, showed that stress, tobacco smoking and malnutrition were first in the rank order. Only 25% of the patients stated a germ or contact with another TB patient as the main cause of the disease whatever the order was.

Analysis of the patients’ tobacco smoking status showed 104 (62.27%) active smokers at the time of diagnosis and the proportion decreased only slightly during the course of therapy.

The majority of patients would inform their family and/or friends about their illness, but one third of them would rather tell nobody.

## Discussion

The original BIPQ, which we implemented in patients with TB, allowed rapid assessment of illness perceptions and took just a few minutes to complete. The patients’ perceptions of the disease varied widely. The over-all BIPQ score is concordant with the clinical TB score at the beginning of treatment, but significantly differed from BIPQ mean values at the end of the initial phase of therapy. This could be expected since higher BIPQ scores indicate a more threatening view of the disease, and higher TBscore indicates more severe clinical presentation. The mean value of the total BIPQ score in the current study is lower than that in a group of patients with chronic obstructive pulmonary disease (COPD) but higher than in those with allergic rhinitis assessed by the same methodology (Pesut et al. 2010; Pesut et al. 2014). This may suggest that TB is perceived as a less threatening disease than COPD. While COPD patients see the BIPQ item, duration, as the most threatening, the results of implementation of the BIPQ in TB patients may reflect positive effects of the efforts to describe TB as a curable disease, i.e. a disease with a defined duration. While a study in Bangladesh showed widespread belief that TB is not curable (Karim et al. 2011), ours revealed that TB patients believed in treatment (the highest and significantly increasing score for treatment control). On the other hand, the mean score for identity (experience of symptoms correctly referred to TB) as one of the lowest BIPQ scores suggests that further efforts are needed to make TB less mysterious and confusing for patients. Thus, TB patients recognized symptoms but were not able to refer them to the illness itself. Furthermore, a highly negative correlation was found between the item, understanding, and clinical score, as well as a significant lack of patients’ knowledge about the main cause of TB. The present findings have implications both for routine clinical practice and for general plans for TB control strategies in terms of an improved approach towards education of patients and the population about TB as an infectious disease and its clinical presentation. A new TBNET project, ExplainTB, has been developed to meet this purpose worldwide (TBNET Tuberculosis Network European Trials Group 2014).

The majority of our TB patients were active smokers at the time of diagnosis and many of them listed smoking as the main cause of the disease. Tobacco smoking is a major and the most preventable cause of morbidity and mortality in the world (World Health Organization 2013; Slama, 2004). It has been shown to decrease both cellular and humoral immunity in humans (Sopori, 2002; Arcavi and Benowitz 2003; Bates et al. 2007). Its association with TB was the focus of several studies, which demonstrated its influence on the severity and clinical course of the disease, and indicated an increased rate of TB relapses in smokers (Bates et al. 2007; Bothamley, 2005). Apart from education on the harmful effects of tobacco smoking and its association with TB, proper professional help in smoking cessation should be offered to those TB patients who are not able to quit smoking alone.

The finding that one third of the patients would not tell anybody about their illness, might suggest that fear of social exclusion still exists (Story et al. 2006) and necessitates further research on stigma in TB (Ahmed Suleiman et al. 2013).

Instead of the long and time consuming Illness Perception Questionnaire - IPQ (or its revised version IPQ-R), we have used the equally valid BIPQ (Broadbent et al. 2006) to record patients’ personal beliefs about TB. An advantage of this approach was that we were able to obtain initial information from almost all patients in successive series, including severely ill ones, thus excluding a potential selection bias. The study design enabled us to obtain a general picture of illness perception in TB quickly and to identify rapidly the particular results of the test for each patient. We could also identify the highest and the lowest BIPQ item scores in the study group, which included double the number suggested to be necessary (Bean et al. 2007). We could not obtain uniform follow up and data collection to maintain all the enrolled patients’ longitudinal data by closure of the study, so there was a decrease in the number patients at the 2-month point. This was not related to their condition but to notification problems and data collection. However, the paired t-test analysis showed increased emotional stability and control over time when the patients apparently gained increased control over their illness, increased believe in the treatment with decreasing symptoms. The emotional response scores were inverse to the illness identity scores and disease control scores at the two time points.

Assessment of illness perception in routine clinical practice could address problems in patients’ behavior that may disturb adherence to treatment and lead to default (Hasker et al. 2008). Since illness perceptions can be changed, the results of our study have potential utility in intervention design and health promotion.

## Conclusion

This study represents the first assessment of illness perception in TB by implementation of the Brief Illness Perception Questionnaire – BIPQ. The 9-item questionnaire allows rapid assessment of illness perception, especially of its cognitive and emotional aspects. Illness perception in TB shows wide variability and a positive correlation with clinical disease score. The results show that TB patients believe in treatment but also indicate further need of education aimed to make TB causes clear and illness identity less confusing. The fact that cohort had a high proportion of smokers that failed to quit smoking during the treatment indicates that more efforts to stop smoking are warranted. Implementation of the BIPQ in routine practice would allow better understanding of patients’ behavior that could lead to default and drug resistance. Further study is needed to investigate the influence of illness perception on treatment outcome in TB.

## Authors’ information

TBNET – Tuberculosis Network European Trials Group contributors are as follows:

Ivan Solovic, Catholic University, Ruzomberk, Slovakia; National Institute for TB, Lung Diseases and Thoracic Surgery, Vysne Hagy, Slovakia, e-mail: solovic@hagy.sk

Katarzyna Kruczak. Jagiellonian University School of Medicine, Kracow, Poland, e-mail: krucza@gmail.com

Raquel Duarte, University of Porto School of Medicine; Chest Disease Centre, Vila Nova de Gaia, Portugal, e-mail: raquelafduarte@gmail.com

Adriana Sorete-Arbore, Hospital of Lung Diseases and TB, Iasi, Romania, e-mail: asnana@mail.dntis.ro

The other contributors:

Marinela Raileanu, Institute of Pneumology "Marius Nasta", Bucharest, Romania, cabuta90@hotmail.com

Irina Strambu, Institute of Pneumology "Marius Nasta", Bucharest, Romania, e-mail: istrambu@yahoo.com

Ljudmila Nagorni-Obradovic, University of Belgrade School of Medicine, Internal Medicine Department, Belgrade, Serbia, e-mail: ljudmila.nagorni@kcs.ac.rs

Tatjana Adzic, University of Belgrade School of Medicine, Internal Medicine Department, Belgrade, Serbia, e-mail: tatjana.adzic@kcs.ac.rs

Zorica Lazic, University Centre Kragujevac, Department of Lung Diseases, Kragujevac, Serbia, e-mail: zokal@eunet.rs

Maria Zlatev-Ionescu, Clinical Hospital of Infectious Diseases "Dr.V.Babes" Pulmonary Diseases, Bucharest, Romania, e-mail: zlatevimaria@yahoo.com

Sorokhaibam Bhagyabati, Regional Research Medical Centre, Manipur, India, drsbdevi@yahoo.com

Irom Ibungo Singh, Regional Research Medical Centre, Manipur, India, e-mail: iisingh2001@yahoo.com

Govind Narayan Srivastava, Baranas Hindu University, Varanasi, India, e-mail: gn_sri@yahoo.co.in

## Electronic supplementary material

Additional file 1: **The Brief Illness Perception Questionnaire.** (DOC 26 KB)

## References

[CR1] Ahmed Suleiman MM, Sahal N, Sodemann M, El Sony A, Aro AR (2013). Tuberculosis stigma in Gezira State, Sudan: a case–control study. Int J Tuberc Lung Dis.

[CR2] Arcavi L, Benowitz NL (2003). Cigarette smoking and infection. Arch Intern Med.

[CR3] Bates MN, Khalakdina A, Pai M, Chang L, Lessa F, Smith KR (2007). Risk of tuberculosis from exposure to tobacco smoke: a systematic review and meta-analysis. Arch Intern Med.

[CR4] Baussano I, Pivetta E, Vizzini L, Abbona F, Bugiani M (2008). Predicting tuberculosis treatment outcome in a low-incidence area. Int J Tuberc Lung Dis.

[CR5] Bean D, Cundy T, Petrie KJ (2007). Ethnic differences in illness perceptions, self-efficacy and diabetes self-care. Psychol Health.

[CR6] Bothamley GH (2005). Smoking and tuberculosis: a chance or causal association?. Thorax.

[CR7] Broadbent E, Petrie KJ, Main J, Weinman J (2006). The Brief Illness Perception Questionnaire (BIPQ). J Psychosom Res.

[CR8] Hasker E, Khodjikhanov M, Usarova S, Asamidinov U, Yuldashova U, van der Werf MJ, Uzakova G, Veen J (2008). Default from tuberculosis treatment in Tashkent, Uzbekistan; who are these defaulters and why do they default?. BMC Infect Dis.

[CR9] Hasker E, Khodjikhanov M, Sayfiddinova S, Rasulova G, Yuldashova U, Uzakova G, Butabekov I, Veen J, van der Werf MJ, Lefevre P (2010). Why do tuberculosis patients default in Tashkent City, Uzbekistan? A qualitative study. Int J Tuberc Lung Dis.

[CR10] Jordan TS, Davies PD (2010). Clinical tuberculosis and treatment outcomes. Int J Tuberc Lung Dis.

[CR11] Karim F, Johansson E, Diwan VK, Kulane A (2011). Community perceptions of tuberculosis: a qualitative exploration from a gender perspective. Public Health.

[CR12] Migliori GB, Lange C, Centis R (2008). Resistance to secondline injectables and treatment outcomes in multidrugresistant and extensively drug-resistant tuberculosis cases. Eur Respir J.

[CR13] Pesut D, Ciobanu L, Bhagyabati S, Nagorni-Obradovic L, Raljevic S, Raileanu M, Bulajic M, Bursuc B, Sepiashvili R (2010). Illness Perception in COPD Patients. Advances in Allergy, Asthma & Immunology: from Basic Science to Clinical Management.

[CR14] Pesut D, Raskovic S, Tomic-Spiric V, Bulajic M, Bogic M, Bursuc B, Peric-Popadic A (2014). Gender differences revealed by the Brief Illness Perception Questionnaire in allergic rhinitis. Clin Respir J.

[CR15] Petrie KJ, Cameron L, Ellis CJ, Buick D, Weinman J (2002). Changing illness perceptions after myocardial infarction: an early intervention randomized controlled trial. Psychosom Med.

[CR16] Petrie KJ, Broadbent E, Meechan G, Cameron LD, Leventhal H (2003). Self-regulatory interventions for improving the management of chronic illness. The Self-Regulation of Health and Illness Behaviour.

[CR17] Raviglione MC, Smith IM (2007). XDR tuberculosis – implications for global public health. N Engl J Med.

[CR18] Slama K (2004). Current challenges in tobacco control. Int J Tuberc Lung Dis.

[CR19] Sopori M (2002). Effects of cigarette on the immune system. Nat Rev Immunol.

[CR20] Stop TB Partnership and World Health Organization (2014). The global plan to stop TB, 2006–2015.

[CR21] Story A, van Hest R, Hayward A (2006). Tuberculosis and social exclusion. BMJ.

[CR22] TBNET (Tuberculosis Network European Trials Group) (2014). Project Explain TB.

[CR23] Wejse C, Gustafson P, Nielsen J, Gomes VF, Aaby P, Andersen PL, Sodemann M (2008). TBscore: Signs and symptoms from tuberculosis patients in a low-resource setting have predictive value and may be used to assess clinical course. Scand J Infect Dis.

[CR24] WHO (2010). WHO/HTM/TB/2009.420. Guidelines for Treatment of Tuberculosis.

[CR25] World Health Organization (2003). Management of Tuberculosis. Training for Health Facility Staff. Module D: Inform Patients About TB.

[CR26] World Health Organization (2011). WHO Progress Report 2011. Towards Universal Access to Diagnosis and Treatment of Multidrug-Resistant and Extensively Drug-Resistant Tuberculosis by 2015.

[CR27] World Health Organization (2013). WHO Report on the Global Tobacco Epidemic 2013.

[CR28] World Health Organization (2014). Global tuberculosis report 2014.

