# Supplementary material for: Illness perception in tuberculosis by implementation of the Brief Illness Perception Questionnaire – a TBNET study
Source: Springerplus. 2014 Nov 8;3:664. doi: 10.1186/2193-1801-3-664 (PMC4233026; doi:10.1186/2193-1801-3-664)
Supplement: Supplementary file 1 — Additional file 1: The Brief Illness Perception Questionnaire. (DOC 26 KB) [file 40064_2014_1357_MOESM1_ESM.doc]

**Additional file – The Brief Illness Perception Questionnaire**

For the following questions, please circle the number that best corresponds to your views:

_________________________________________________________________________

1. How much does your illness affect your life?

0 1 2 3 4 5 6 7 8 9 10

no affect severely

at all affects my life

2. How long do you think your illness will continue?

0 1 2 3 4 5 6 7 8 9 10

a very forever

short time

3. How much control do you feel you have over your illness?

0 1 2 3 4 5 6 7 8 9 10

absolutely extreme amount

no control of control

4. How much do you think your treatment can help your illness?

0 1 2 3 4 5 6 7 8 9 10

not at all extremely

helpful

5. How much do you experience symptoms from your illness?

0 1 2 3 4 5 6 7 8 9 10

no symptoms many severe

at all symptoms

6. How concerned are you about your illness?

0 1 2 3 4 5 6 7 8 9 10

not at all extremely

concerned concerned

7. How well do you feel you understand your illness?

0 1 2 3 4 5 6 7 8 9 10

don’t understand

understand very clearly

at all

8. How much does your illness affect you emotionally? (e.g. does it make you angry, scared, upset or depressed?)

0 1 2 3 4 5 6 7 8 9 10

not at all extremely

affected affected

emotionally emotionally

9. Please list in rank-order the three most important factors that you believe caused your illness.

The most important causes for me:

1. ___________________________2. _________________________3. __________________________
